# Exploring Representation of Diverse Samples in fMRI Studies Conducted in Patients With Cardiac-Related Chronic Illness: A Focused Systematic Review

**DOI:** 10.3389/fnhum.2020.00108

**Published:** 2020-05-14

**Authors:** Lenette M. Jones, Emily Ginier, Joseph Debbs, Jarrod L. Eaton, Catherine Renner, Jaclynn Hawkins, Rosanna Rios-Spicer, Emily Tang, Catherine Schertzing, Bruno Giordani

**Affiliations:** ^1^School of Nursing, University of Michigan, Ann Arbor, MI, United States; ^2^Taubman Health Sciences Library, University of Michigan, Ann Arbor, MI, United States; ^3^School of Social Work, University of Michigan, Ann Arbor, MI, United States; ^4^Psychiatry, Neurology, Psychology, and Nursing, University of Michigan, Ann Arbor, MI, United States

**Keywords:** fMRI, cardiovascular disease, sample demographics, health disparities, chronic illness

## Abstract

**Introduction/Purpose:** Cardiovascular disease (CVD) is the leading cause of death worldwide, and in the United States alone, CVD causes nearly 840,000 deaths annually. Using functional magnetic resonance imaging (fMRI), a tool to assess brain activity, researchers have identified some brain-behavior connections and predicted several self-management behaviors. The purpose of this study was to examine the sample characteristics of individuals with CVD who participated in fMRI studies.

**Methods:** A literature search was conducted in PubMed, CINAHL, and Scopus. No date or language restrictions were applied and research methodology filters were used. In October 2017, 1659 titles and abstracts were identified. Inclusion criteria were: (1) utilized an empirical study design, (2) used fMRI to assess brain activity, and (3) focused on patients with CVD-related chronic illness. Articles were excluded if they: were theory or opinion articles, focused on mental or neuropathic illness, included non-human samples, or were not written in English. After duplicates were removed (230), 1,429 titles and abstracts were reviewed based on inclusion criteria; 1,243 abstracts were then excluded. A total of 186 studies were reviewed in their entirety; after additional review, 142 were further excluded for not meeting the inclusion criteria. Forty-four articles met criteria and were included in the final review. An evidence table was created to capture the demographics of each study sample.

**Results:** Ninety eight percent of the studies did not report the racial or ethnic composition of their sample. Most studies (66%) contained more men than women. Mean age ranged from 38 to 78 years; 77% reported mean age ≥50 years. The most frequently studied CVD was stroke (86%), while hypertension was studied the least (2%).

**Conclusion:** Understanding brain-behavior relationships can help researchers and practitioners tailor interventions to meet specific patient needs. These findings suggest that additional studies are needed that focus on populations historically underrepresented in fMRI research. Researchers should thoughtfully consider diversity and purposefully sample groups by including individuals that are: women, from diverse backgrounds, younger, and diagnosed with a variety of CVD-related illnesses. Identifying and addressing these gaps by studying more representative samples will help healthcare providers reduce disparities and tailor interventions for all CVD populations.

## Introduction

According to the World Health Organization, cardiovascular disease (CVD) is an umbrella term which includes a number of heart and blood vessel disorders. Disorders include cerebrovascular disease (stroke) and hypertension (World Health Organization). About 121.5 million Americans are living with some form of CVD, with direct and indirect costs of total cardiovascular diseases and stroke totaling more than $351.2 billion (Benjamin et al., 2018, 2019). CVD is currently the leading cause of death in the U.S., causing nearly 836,546 deaths annually (Benjamin et al., 2018, 2019). In addition to its high mortality rate, CVD is associated with other chronic illnesses, such as end-stage renal disease and diabetes (Liu et al., 2014; Leon and Maddox, 2015). Groups that differ by race, ethnicity, education level, gender, and socioeconomic status are negatively and disproportionately affected by CVD and other chronic illnesses, and trends show that the gaps in these disparities are widening (Di Chiara et al., 2015; Havranek et al., 2015; Singh et al., 2015; Mehta et al., 2016). Initiatives that target specific social determinants of health are needed (Valero-Elizondo et al., 2018).

Some CVD-related illnesses, such as hypertension, can be controlled with lifestyle modifications such as consistently eating a healthy diet, engaging in regular physical activity, and adhering to antihypertensive medication (Nicolson et al., 2004). Such activities are often referred to as self-management behaviors. To improve upon these self-management behaviors, and therefore reduce associated risks with CVD, more studies are needed to assist practitioners to better guide patients toward consistent healthy behaviors. As such, studies that link brain activity via functional magnetic resonance imaging to self-management behaviors may establish an important foundation in achieving desirable patient outcomes.

Functional magnetic resonance imaging, or fMRI, is a tool that measures brain activity by detecting changes in blood oxygenation and flow that correspond to neural activity (Devlin et al., 2007). The brain increases oxygen demand in areas that are more active, and to meet this demand, blood flow increases to the area. In previous studies, researchers have used fMRI to predict self-management behaviors, such as sunscreen use and smoking cessation (Falk et al., 2010, 2011). Falk et al. (2010) measured neural activity in the medial prefrontal cortex (MPFC) of the brain while people watched persuasive messages about the value of using sunscreen regularly (Falk et al., 2010). They used these measurements to predict whether individuals would increase their sunscreen use, above and beyond self-report. They found that activity in the MPFC was significantly related to persuasion-induced behavior change, or increased sunscreen use, over the course of two weeks (Falk et al., 2010).

A subsequent study looked at the same area of the brain (MPFC) and tested whether neural activity in response to messages promoting smoking cessation could predict smoking cessation, above and beyond self-report (Falk et al., 2011). The researchers found that increases in MPFC activity were associated with decreases in expired carbon monoxide following exposure to professionally developed quitting ads (Falk et al., 2011). In both studies, by measuring MPFC activity while subjects viewed the persuasive messages, the researchers were able to predict the behavioral efficacy of the messages “above and beyond what participants' own self-reported attitude and intention change could predict” (Falk et al., 2010, 2011).

Other studies have examined the antagonistic relationship between analytic and socio-emotional neuroprocessing. Jack et al. (2013) found that individuals who were better able to process both analytic and socioemotional prompts, were better able to make plans, and act on the plans that they had developed (Jack et al., 2013). The analytic network, also known as the task positive network, pertains to skills, problem solving, and goal-directed actions (Duncan and Owen, 2000; Jack et al., 2013). Thus, it is activated by attention-demanding tasks (Fox et al., 2005; Uddin et al., 2009; Bressler and Menon, 2010). By contrast, the empathetic network, also referred to as the default mode network, encompasses emotional management and self-awareness, and is activated during periods of wakeful rest (Denny et al., 2012; Eisenberger and Cole, 2012; Marstaller et al., 2016).

Analytic information and emotional information are processed in different areas of the brain and are anti-correlated (Jack et al., 2013). The analytic information is processed in prefrontal and parietal areas of the brain, while the emotional information is processed in the posterior cingulate and medial prefrontal cortices (Duncan and Owen, 2000; Fox et al., 2005). This means that responses to different types of information varies depending on individual characteristics (Singh et al., 2015). One study reported findings that socio-emotional processing was positively associated with sharing of health information with others (Jones et al., 2019). Better understanding how the brain processes different types of information will help to develop individualized, and potentially more effective self-management interventions.

The purpose of this systematic review was to examine the demographic characteristics presented in fMRI studies that have been conducted with patients with CVD-related illnesses. Specifically, we aimed to evaluate studies on brain activity in participants with CVD to determine which patient populations the findings were applicable to. Results from the studies reviewed in this paper can be used to guide future studies to explore brain activity patterns to predict specific behaviors. For example, fMRI studies that focus on patients with CVD would be useful in helping researchers and clinicians better understand how brain activity patterns can be used to predict self-management of CVD, and which interventions may be more useful to individuals with certain patterns of brain activity (Moore et al., 2019).

## Methods

### Team

The team that conducted this work consisted of a doctorally-prepared nurse scientist, a health sciences librarian, and four undergraduate students. The Preferred Reporting Items for Systematic Reviews and Meta-Analyses (PRISMA) method was used to guide reporting for this review.

### Search Strategy

The databases PubMed, CINAHL, and Scopus were searched on October 2, 2017 to retrieve articles on use of functional magnetic resonance imaging (fMRI) in populations with chronic illnesses (see Appendix A). Controlled vocabulary (i.e., Medical Subject Headings and CINAHL Headings) and keywords were used to identify related terms for chronic illness and functional magnetic resonance imaging (fMRI). No date or language restrictions were applied to the search. In order to limit retrieval to treatment and diagnostic studies that contain empirical evidence, therapy, and diagnosis research methodology filters were used in PubMed and adapted for use in CINAHL and Scopus (Lokker et al., 2011). A total of 1,659 titles and abstracts were identified for review.

### Study Selection

Two reviewers from the study team independently assessed study eligibility using Covidence systematic review software [Covidence Systematic Review Software]. Studies were selected for further review if they met the following criteria: (1) utilized an empirical study design, (2) used fMRI to assess brain activity, and (3) focused on patients with CVD-related chronic illness.

Articles were excluded that were not original research (e.g., theory or opinion articles), focused on mental or neuropathic illness, included non-human samples, or were not written in English.

### Search Results

**Figure 1:**
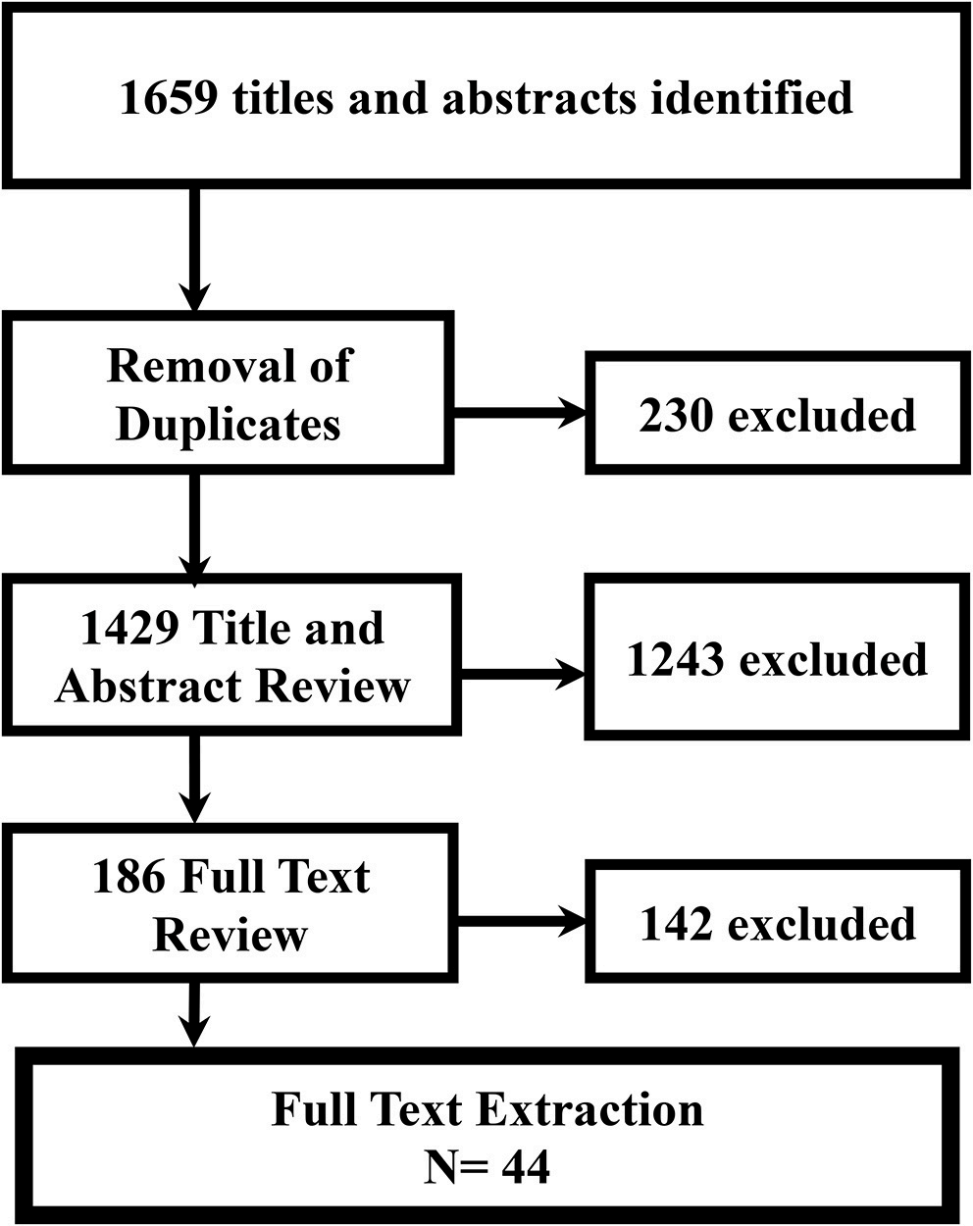
Study eligibility flow diagram.

The search strategies retrieved a total of 1,659 articles. After the removal of duplicates, 1,429 articles remained for screening, of which 1,243 were excluded at the title and abstract level. Following a full-text review of 186 articles, 142 were excluded for not meeting criteria. Forty-four articles were included in the final review (see Figure 1 for a flow diagram).

### Presentation of Results

A table that categorized the studies based on geographical location was created to examine the variety of settings in which the studies had been conducted (see Table 1). Another table was developed to provide brief quantitative and descriptive summaries of the studies' design, gender distribution, mean age, and brain regions of interests (see Table 2). Lastly, a third table was created to determine which demographic characteristics (race, age, gender, income, and education) were examined in each study sample (see Table 3). The studies were categorized by the first author's last name and year, the chronic illness being studied, and the number of participants in each sample.

**Table 1:** Geographic location.

**Chronic illness**	**Location**	**# of studies**
Stroke	USA	15
	Germany	4
	Korea	3
	United Kingdom	3
	Austria	2
	Canada	2
	Czech Republic	2
	Brazil	1
	China	1
	Finland	1
	France	1
	Italy	1
	Japan	1
	Netherlands	1
CKD/ESRD	China	2
	Germany	1
	USA	2
Type 2 DM	China	1
HTN	USA	1

*CKD, chronic kidney disease; ESRD, end-stage renal disease; DM, diabetes mellitus; HTN, hypertension*.

**Table 2:** Summary of studies.

**Chronic illness**	**# of studies**	**Study design**		**Mean age (years)**		**Gender distribution**	**Brain regions of interest**
Stroke	38	32 RCTs	6 cohort	Lowest mean age reported: 40 (range = 34,67)	Highest mean age reported: 73 (SD = 4)	Males > females	•Areas of chronic diaschisis or peristroke areas •Primary motor cortex •Perilesional tissue •Supplementary motor area •Posterior cerebellar lobe
CKD/ESRD	4		4 cohort	Lowest mean age reported: 34 (SD = 7)	Highest mean age reported: 72 (SD = 7)	Males > females	•Default mode network •Hippocampus •Frontal and parietal lobes •Bilateral inferior frontal gyrus •Right superior temporal gyrus
Type 2 diabetes	1		Cohort	41 (range = 31, 53)		Males > females	•Anterior cingulate cortex •Bilateral DLPFC
Hypertension	1		Cohort	67 (SD = 8.9)		Males < females	•Frontal and medial •Temporal lobes

*CKD, chronic kidney disease; ESRD, end-stage renal disease; RCT, randomized controlled trial*.

**Table 3:** Study characteristics.

	**Chronic illness**	**# of participants**	**Demographic characteristic reported**				
**References**			**Race**	**Age**	**Gender**	**Income**	**Education**
Brownsett et al. (2014)	Stroke	16	–	+	+	–	+
Carey et al. (2002)	Stroke	10	–	+	+	–	–
Deng et al. (2012)	Stroke	16	–	+	+	–	–
Fair et al. (2009)	Stroke	6	–	–	–	–	–
Gold et al. (2005)	Hypertension	54	–	+	–	–	–
He et al. (2015)	Type 2 diabetes	24	–	+	+	–	+
Hodgson et al. (2014)	Stroke	16	–	+	+	–	+
Jahanian et al. (2014)	CKD	20	–	+	+	–	–
Kato et al. (2002)	Stroke	11	–	+	+	–	–
Kielar et al. (2016)	Stroke	38	–	+	+	–	+
Kim et al. (2006)	Stroke	18	–	+	+	–	–
Kimberley et al. (2004)	Stroke	16	–	+	+	–	–
Kononen et al. (2012)	Stroke	11	–	+	+	–	–
Kwon et al. (2007)	Stroke	31	–	+	+	–	–
Landsmann et al. (2016)	Stroke	24	–	+	+	–	+
Lazaridou et al. (2013)	Stroke	17	–	–	–	–	–
Li et al. (2016)	ESRD	51	–	+	+	–	+
Luft et al. (2008)	Stroke	71	+	+	+	–	–
Luft et al. (2004a)	Stroke	21	–	+	+	–	–
Luft et al. (2004b)	Stroke	28	–	+	+	–	–
Lux et al. (2010)	ESRD	24	–	+	+	–	+
Mattioli et al. (2014)	Stroke	12	–	+	+	–	+
Meehan et al. (2011)	Stroke	18	–	+	+	–	–
Menke et al. (2009)	Stroke	8	–	+	+	–	–
Michielsen et al. (2011)	Stroke	40	–	+	+	–	–
Milot et al. (2014)	Stroke	20	–	+	+	–	–
Pelicioni et al. (2016)	Stroke	21	–	+	+	–	–
Pineiro et al. (2001)	Stroke	28	–	+	+	–	–
Pinter et al. (2013)	Stroke	7	–	+	–	–	–
Ramos-Murguialday et al. (2013)	Stroke	32	–	+	+	–	–
Rehme et al. (2015)	Stroke	21	–	+	+	–	–
Rijntjes et al. (2011)	Stroke	12	–	+	+	–	–
Schaechter et al. (2007)	Stroke	7	–	+	+	–	–
Schaechter et al. (2002)	Stroke	4	–	+	+	–	–
Shin et al. (2008)	Stroke	14	–	+	+	–	–
Sun et al. (2013)	Stroke	18	–	+	+	–	–
Szaflarski et al. (2011)	Stroke	8	–	+	+	–	+
Takahashi et al. (2008)	Stroke	13	–	+	+	–	–
Tombari et al. (2004)	Stroke	18	–	+	+	–	–
Veverka et al. (2014)	Stroke	14	–	+	+	–	–
Veverka et al. (2012)	Stroke	14	–	+	+	–	–
von Lewinski et al. (2009)	Stroke	9	–	+	+	–	–
Whitall et al. (2011)	Stroke	111	–	+	+	–	–
Zhang et al. (2015)	ESRD	46	–	+	+	–	+

*CKD, chronic kidney disease; ESRD, end-stage renal disease*.

## Results

### Cardiovascular-Related Diseases

There were 38 studies that focused on patients that had strokes (86%) (Pineiro et al., 2001; Carey et al., 2002; Kato et al., 2002; Schaechter et al., 2002, 2007; Kimberley et al., 2004; Luft et al., 2004a,b, 2008; Tombari et al., 2004; Kim et al., 2006; Kwon et al., 2007; Shin et al., 2008; Takahashi et al., 2008; Fair et al., 2009; Menke et al., 2009; von Lewinski et al., 2009; Meehan et al., 2011; Michielsen et al., 2011; Rijntjes et al., 2011; Szaflarski et al., 2011; Whitall et al., 2011; Deng et al., 2012; Kononen et al., 2012; Veverka et al., 2012, 2014; Lazaridou et al., 2013; Pinter et al., 2013; Ramos-Murguialday et al., 2013; Sun et al., 2013; Brownsett et al., 2014; Hodgson et al., 2014; Mattioli et al., 2014; Milot et al., 2014; Rehme et al., 2015; Kielar et al., 2016; Landsmann et al., 2016; Pelicioni et al., 2016). Of the 38 stoke studies, the majority (84%) of the studies were randomized controlled trials (see Table 2). Four cohort studies (9%) had participants with chronic kidney disease (Lux et al., 2010; Jahanian et al., 2014; Zhang et al., 2015; Li et al., 2016). One cohort study had participants who had been diagnosed with type 2 diabetes (2%) and one cohort study had participants who had been diagnosed with hypertension (2%) (Gold et al., 2005; He et al., 2015).

### Race, Ethnicity, and Geographic Location

The vast majority of studies (98%) did not report the racial or ethnic composition of the sample (Table 3). In the one study that did examine race, the report stated that over half of the participants were “Black,” followed by “White” participants, and a small percentage of “Hispanic/Other” participants (Luft et al., 2008). Most of the stroke studies were conducted in the United States (Table 1). Half of the renal studies were conducted in China. The type 2 diabetes study was conducted in China. The hypertension study was conducted in the United States. See Table 1 for additional details on where the studies were conducted.

### Age

The majority (95%) of the studies provided the age of the participants (Table 3). There was variation in the manner which age was presented in each study. Some studies provided an overall mean for all of the participants, while others reported means for the intervention or affected groups compared to the control or “healthy” groups. The mean age of the participants ranged from 34 to 73 years; 77% of the studies reported mean age ≥50 years.

### Gender

The majority (91%) of the studies reported the gender distribution of the sample. Most studies (66%) contained more men than women (Table 2). When examining the samples by chronic illness, studies focused on stroke patients had more men than women. Studies of chronic renal patients had more men than women. The study of patients with type 2 diabetes had more men than women, while the study of participants with hypertension had more women than men.

### Income

None of the studies included in this review presented information on the participants' income.

### Education

A total of 10 studies (23%) provided information on the participants' education levels, overall (Table 3). In terms of stroke studies specifically, six out of 38 studies (16%) reported on the education of participants. The mean years of education ranged from 6 to 16.8 years for five of the studies. An additional study reported that four of its participants received 14–17 years of education, however, no education data was available for the remainder of the participants. With regards to chronic kidney disease, three out of the four studies (75%) reported on the education of participants. The mean education ranged from 11.4 to 13.1 years. Regarding the sole type 2 diabetes study, mean participant education was reported as 9.8 years. Lastly, the singular hypertension study included in this review did not include information with respect to years of education of participants.

## Discussion

The objective of this study was to examine the sample characteristics of individuals with CVD who participated in fMRI studies. A total of 44 studies were included in the review. Our findings demonstrate that race and ethnicity and socioeconomic status of participants are not often considered, as demonstrated in the inconsistency in reporting demographic characteristics from across the studies. This review highlights the need for more stringent and detailed collection of demographic data from participants enrolled in fMRI studies. Additional reviews are needed that evaluate fMRI studies sample sizes and stricter statistical threshold. Future studies are needed that focus on populations that have been historically underrepresented in fMRI/CVD-related research. Only one study mentioned the racial or ethnic composition of participants. Additionally, in 66% of the studies, the majority of participants were male. By studying a sample that is more representative of the general population and by expanding the type of CVD studied, researchers can identify practices that are relevant for populations that are disproportionately impacted by hypertension, such as African Americans. Diverse populations (i.e., racial and ethnic, gender, and age groups) vary greatly with respect to health risks such as CVD, as well as access to health care and other health disparities (Leigh et al., 2016). Patients with lower incomes and education levels may increased CVD risk (Marshall et al., 2015; Khaing et al., 2017). Additional research is needed to explore differences in brain activity patterns and related behaviors among diverse patients with CVD.

Of the studies identified as having reported CVD-related outcomes, 84% examined stroke, while only 2% examined hypertension or type 2 diabetes. It is important to note that understanding brain-behavior relationships has the potential to help researchers and practitioners tailor interventions to meet specific patient needs. These points further demonstrate a need for additional studies that use fMRI to better understanding brain-behavior relationships among patients with specific CVD-related diseases.

## Limitations

The primary limitation of this study was the variety in reporting of demographic characteristics across the studies. As a result, it limited the summaries and conclusions that could be made. However, this lack of reporting supports the idea that future fMRI research needs to consider and prioritize racial/ethnic background, income, and education during the recruitment and sampling process. Additionally, the original goal of this literature review was to examine the fMRI studies that had be conducted with participants who self-identified as African American and were diagnosed with hypertension. Given that only one study met these criteria, the search strategy was expanded to include studies with conditions associated with hypertension (stroke, ESRD, diabetes). Given the large number of stroke studies in this review, a meta-analysis of these studies would be an interesting contribution to the literature.

## Conclusion

This review suggests that certain groups with CVD disease (women, younger adults, racial/ethnicity minorities) are underrepresented in fMRI research. Therefore, there is a knowledge gap with respect to evidence about brain-behavior connections in groups that are of different races, ethnicities, or genders. Researchers should consider diversity when selecting sampling methods to include individuals from underrepresented groups, such as: women, individuals from diverse backgrounds, younger adults (age <50 years), and those diagnosed with hypertension. When recruiting participants with CVD for fMRI studies, researchers need to consider barriers that prevent these populations from participating, such as socioeconomic status, distrust of the scientific community, cultural barriers, and lack of knowledge related to fMRI research. Identifying and addressing these gaps will lead to the reduction of disparities in fMRI research and improve interventions for all CVD populations.

## Author Contributions

We are pleased to submit this manuscript, entitled Exploring Representation of Diverse Samples in fMRI Studies Conducted in Patients with Cardiac-Related Chronic Illness: A Focused Systematic Review to be considered for publication. This paper highlights findings of a focused review on the demographics of patients with cardiovascular disease who participated in fMRI studies. This manuscript has not been published and is not under submission elsewhere. There are no conflict of interests that exist. All authors contributed substantively to the content of this manuscript and are in agreement for its readiness to be considered for publication: LJ and EG: development/implementation of methods, study review, and manuscript preparation. JD, JE, CR, and BG: manuscript preparation. JH: study review and manuscript preparation. RR-S: implementation of methods and manuscript preparation. ET and CS: implementation of methods, study review, and manuscript preparation.

## Conflict of Interest

The authors declare that the research was conducted in the absence of any commercial or financial relationships that could be construed as a potential conflict of interest.

## Appendix A

### Search Strategies by Database

#### PUBMED

#1. (“fMRI” OR “functional magnetic resonance imaging” OR “functional MRI” OR ((“brain imaging” OR neuroimag^*^) AND functional) OR neuroprocess^*^).

#2. (“Chronic Disease”[Mesh] OR chronically OR chronic).

#3. ((sensitiv^*^[Title/Abstract] OR sensitivity and specificity[MeSH Terms] OR diagnose[Title/Abstract] OR diagnosed[Title/Abstract] OR diagnoses[Title/Abstract] OR diagnosing[Title/Abstract] OR diagnosis[Title/Abstract] OR diagnostic[Title/Abstract] OR diagnosis[MeSH:noexp] OR diagnostic ^*^ [MeSH:noexp] OR diagnosis,differential[MeSH:noexp] OR diagnosis[Subheading:noexp]) OR ((clinical[Title/Abstract] AND trial[Title/Abstract]) OR clinical trials as topic[MeSH Terms] OR clinical trial[Publication Type] OR random^*^[Title/Abstract] OR random allocation[MeSH Terms] OR therapeutic use[MeSH Subheading])).

#4. #1 AND #2 AND #3.

#### CINAHL

S1. (“fMRI” OR “functional magnetic resonance imaging” OR “functional MRI” OR ((“brain imaging” OR neuroimag^*^) AND functional) OR neuroprocess^*^).

S2. (MH “Chronic Disease” OR chronically OR chronic).

S3. ((TI sensitiv^*^ OR AB sensitiv^*^ OR MH “Sensitivity and Specificity” OR TI diagnose OR AB diagnose OR TI diagnosed OR AB diagnosed OR TI diagnoses OR AB diagnoses OR TI diagnosing OR AB diagnosing OR TI diagnosis OR AB diagnosis OR TI diagnostic OR AB diagnostic OR MH “Diagnosis” OR MH “Diagnostic Imaging” OR MH “Diagnostic Services” OR MH “Diagnostic Errors” OR MH “Diagnosis, Differential” OR MW DI) OR (((TI clinical OR AB clinical) AND (TI trial OR AB trial)) OR MH “Clinical Trials+” OR TI random^*^ OR AB random^*^ OR MH “Random Assignment” OR MW TH)).

S4. S1 AND S2 AND S3.

#### SCOPUS

#1. TITLE-ABS-KEY(“fMRI” OR “functional magnetic resonance imaging” OR “functional MRI” OR ((“brain imaging” OR neuroimag^*^) AND functional) OR neuroprocess^*^).

#2. TITLE-ABS-KEY (chronic OR chronically).

#3. TITLE-ABS-KEY (cardiovascular OR CVD OR heart OR circulatory OR myocardial OR myocardium OR “blood vessel” OR hypertension OR hypertensive OR “blood pressure”).

#4. ALL(sensitiv^*^ OR Specificity OR diagnose OR diagnosed OR diagnoses OR diagnosing OR diagnosis OR diagnostic OR (clinical AND trial^*^) OR random^*^ OR “therapeutic use”).

#5. #1 AND #2 AND #3 AND #4.
